# Enhanced differentiation of breast lesions through integration of microvascular flow imaging and machine learning algorithms

**DOI:** 10.3389/fonc.2026.1827050

**Published:** 2026-06-17

**Authors:** Fangfang Zhou, Wanling Lin, Jiqin Yao, Xiaoxi Lu, Lifang Yu

**Affiliations:** 1 Department of Ultrasound, Hangzhou Women’s Hospital (Hangzhou Maternal and Child Health Care Hospital), Hangzhou, Zhejiang, China; 2 Department of Ultrasound, Affiliated Hangzhou First People’s Hospital, School of Medicine, Westlake University, Hangzhou, Zhejiang, China

**Keywords:** blood flow grading, breast tumor, machine learning model, microvascular flow imaging (MV-Flow), vascular index

## Abstract

**Introduction:**

Breast cancer diagnosis relies on imaging, yet conventional Doppler ultrasound possesses limitations in visualizing tumor microvasculature. This study aimed to compare Microvascular Flow imaging (MV-Flow) with Color Doppler Flow Imaging (CDFI) and evaluate a machine learning (ML) framework integrating MV-Flow parameters for differentiating breast lesions.

**Methods:**

In this prospective study, 101 breast lesions from 79 patients underwent grayscale ultrasound, CDFI, and MV-Flow. Adler grading and the quantitative Vascular Index (VI) were obtained. Based on six features (age, size, CDFI/MV-Flow Adler grades, VI, BI-RADS), eight ML models were trained (80% data) and tested (20% data). Model performance was evaluated, and SHAP analysis identified key predictors.

**Results:**

MV-Flow demonstrated superior sensitivity, detecting blood flow in 16 lesions missed by CDFI, and showed significantly higher inter-observer agreement (weighted Kappa=0.68 vs. 0.51 for CDFI). The median Vascular Index (VI) was markedly higher in malignant lesions (20.25) compared to benign ones (3.10, P<0.001). The diagnostic AUC for MV-Flow Adler grade, VI alone, and their combination were 0.874, 0.823, and 0.888, respectively. Among eight machine learning models trained on six clinical and sonographic features, the K-Nearest Neighbors model achieved the best performance on the independent test set with an accuracy of 0.927 and an F1-score of 0.947. SHAP analysis identified BI-RADS category and patient age as the most important predictive features in the model.

**Conclusion:**

MV-Flow outperforms CDFI in depicting breast tumor microvasculature. ML models integrating MV-Flow parameters can optimize diagnostic accuracy, offering an objective tool for clinical decision support.

## 1 Introduction

In recent years, the global burden of breast cancer has escalated at an alarming pace, with surging incidence and mortality rates posing a severe and growing threat to women’s health (1). As documented in the 2022 Global Cancer Statistics, breast cancer ranks as the most prevalent malignancy diagnosed in females and persists as a leading contributor to cancer-associated mortality worldwide, with its epidemiological impact continuing to intensify across regions (1). Despite this pressing public health concern, efficacious etiological prevention strategies for breast cancer remain notably elusive. In this context, imaging-based diagnostic modalities have emerged as cornerstones of early detection and therapeutic stratification, exerting a pivotal influence on patient prognosis and clinical outcomes (2). Among the array of available imaging techniques, diagnostic ultrasound stands out for its unique advantages: real-time imaging capability, complete absence of ionizing radiation exposure, and insensitivity to breast tissue density. Extensive clinical investigations have corroborated that the integration of ultrasound into breast imaging protocols substantially elevates the detection sensitivity for small-sized, invasive, and lymph-node-negative breast carcinomas, cementing its status as a first-line screening and diagnostic tool in clinical practice across numerous countries (3). Particularly, ultrasound demonstrates unparalleled diagnostic efficacy in women with dense breast tissue, proficiently identifying occult tumors that evade detection via mammography (4). This attribute is of paramount clinical significance, given that dense breast tissue constitutes an independent risk factor for breast cancer development, and ultrasound’s superior sensitivity directly addresses this critical diagnostic gap (5). Moreover, the non-invasive, real-time nature and radiation-free profile of ultrasound render it a safe, cost-effective, and highly accessible diagnostic modality in routine clinical settings. In contrast to conventional mammography, ultrasound circumvents the risks associated with ionizing radiation, thereby minimizing cumulative radiation exposure for patients undergoing serial screenings (6). Beyond morphological assessment, ultrasound further yields functional insights by quantifying tissue acoustic parameters such as sound velocity and attenuation coefficients, which provide valuable adjunctive information for evaluating lesion stiffness and distinguishing pathological characteristics (7). This real-time functional imaging capability underpins ultrasound’s indispensable role in guiding image-directed biopsies and facilitating precise clinical decision-making.

Breast cancer is characterized as a vascular-dependent neoplasm, wherein intratumoral angiogenesis serves as an indispensable prerequisite for tumor proliferation, local invasion, and distant metastasis (8). Color Doppler Flow Imaging (CDFI) has long been the gold-standard ultrasound technique for evaluating tumor vascularity in clinical practice. Nevertheless, inherent technical constraints of CDFI result in the concurrent filtering of background clutter and low-velocity blood flow signals; consequently, this modality is only capable of visualizing macro-vessels with a diameter exceeding 0.2 mm and relatively high flow velocities, thus failing to accurately reflect the intricate microcirculatory networks within tumors (9). Microvascular Flow Imaging (MV-Flow) represents a paradigm shift in vascular ultrasound imaging, offering enhanced sensitivity and spatial resolution for detecting microcirculatory and low-velocity blood flow components while effectively suppressing clutter artifacts. Furthermore, MV-Flow enables quantitative analysis of vascularity within a predefined region of interest (ROI), generating a quantitative Vascular Index (VI) that serves as an objective biomarker for assessing lesion aggressiveness and differentiating benign from malignant breast lesions (10). A seminal comparative study evaluated the diagnostic efficacy of contrast-enhanced ultrasound combined with micro-flow imaging (CEUS-MFI), standalone MFI, and conventional CDFI in distinguishing benign from malignant breast lesions (11). The findings revealed that CEUS-MFI outperformed both MFI and CDFI in terms of imaging resolution and diagnostic accuracy, with its superior ability to delineate microvascular architectures emerging as a key differentiator (11). Complementary evidence from another clinical trial further substantiated that MFI and its advanced iteration, high-definition micro-flow imaging (HD-MFI), exhibit significantly higher sensitivity than CDFI in detecting intralesional blood flow signals and penetrating micro-vessels, thereby underscoring the immense clinical potential of MV-Flow technology in breast tumor assessment (12).

Contemporary ultrasound-based breast tumor assessment is undergoing a transformative shift, evolving from a paradigm reliant on subjective physician experience and single-parameter morphological analysis toward a holistic, multi-parameter integrated evaluation framework. Although the Breast Imaging Reporting and Data System (BI-RADS) classification—currently the most widely adopted diagnostic lexicon—incorporates both morphological and vascular features, its assessment of tumor vascularity predominantly relies on semi-quantitative methods such as the Adler grading system. This subjectivity inherently introduces substantial inter-observer variability, thereby compromising the reproducibility and reliability of diagnostic interpretations (13). Against this backdrop, a critical knowledge gap persists: how to optimally integrate the rich, quantitative vascular information derived from MV-Flow technology with clinical and conventional ultrasound parameters to maximize diagnostic performance, a question that demands rigorous empirical investigation. Concurrently, artificial intelligence (AI) has emerged as a disruptive force in medical imaging, with machine learning (ML) models demonstrating exceptional proficiency in processing high-dimensional, complex nonlinear datasets. These models hold great promise for overcoming the limitations of traditional statistical approaches and single-parameter assessments by fusing multi-modal features to achieve more objective and accurate differential diagnosis (14, 15). For instance, ML algorithms have already exhibited remarkable diagnostic efficacy in aiding the interpretation of CT images for lung nodule characterization and dermoscopic images for cutaneous lesion diagnosis (16, 17). Thus, the synergistic integration of advanced vascular imaging technologies with cutting-edge intelligent algorithms may pave the way for a novel diagnostic paradigm, addressing the current bottlenecks in ultrasound-based breast cancer diagnosis.

In light of the aforementioned challenges and opportunities, the present prospective study is designed to systematically compare the diagnostic utility of MV-Flow and CDFI in visualizing the microvascular architecture of breast tumors and differentiating benign from malignant lesions. A particular focus is placed on evaluating the diagnostic performance of MV-Flow, with emphasis on inter-observer consistency and the clinical value of its quantitative parameter, the Vascular Index (VI). Furthermore, this study innovatively constructs and validates multiple machine learning models to explore the optimal integration strategy encompassing clinical covariates (age), conventional ultrasound features (BI-RADS classification, tumor size), and advanced hemodynamic parameters (Adler grade derived from CDFI and VI derived from MV-Flow). The overarching goal is to establish a more objective, accurate, and reproducible diagnostic framework, thereby furnishing novel, robust evidence and practical solutions to advance the precision of ultrasound-based breast tumor diagnosis.

## 2 Materials and methods

### 2.1 General information

A prospective cohort of patients who underwent grayscale ultrasound, Color Doppler Flow Imaging (CDFI), and Microvascular Flow Imaging (MV-Flow) examinations at our institution between January 2024 and October 2025 was consecutively enrolled.

1. Inclusion criteria: ① Female patients aged 18 years or older; ② Breast lesions categorized as Breast Imaging Reporting and Data System (BI-RADS) 3, 4, or 5 on ultrasound, with definitive diagnoses confirmed by percutaneous biopsy or surgical pathology; lesions without immediate pathological confirmation were required to complete a 12-month imaging follow-up; ③ Lesions with a maximum diameter ranging from 0.5 cm to 5.0 cm; ④ Voluntary participation of patients with signed written informed consent.
2. Exclusion criteria: ① Lactating or pregnant women; ② A history of prior therapeutic interventions targeting the index lesion, including percutaneous biopsy, surgical resection, radiotherapy, chemotherapy, endocrine therapy, or targeted therapy; ③ Suboptimal ultrasound image quality precluding reliable morphological and hemodynamic assessment; ④ Lack of definitive pathological outcomes or failure to complete the 12-month follow-up protocol.

A total of 79 patients harboring 101 breast lesions were ultimately included in the study cohort, among whom 61 cases presented with solitary lesions and 18 cases with multiple lesions.

### 2.2 Instruments and examination protocols

#### 2.2.1 Ultrasound equipment

All ultrasound examinations were performed using a Samsung Medison R10 ultrasound system equipped with a LA2-14A linear transducer, operating at a frequency range of 2–14 MHz.

#### 2.2.2 Ultrasound examination procedures

All patients underwent sequential grayscale ultrasound, CDFI, and MV-Flow examinations, which were independently conducted by a single radiologist with extensive expertise in breast ultrasound diagnostics and specialized training in MV-Flow technology. During the examination, patients were positioned in the supine position with both arms elevated to fully expose the breast and axillary regions. The system was initialized to the dedicated breast imaging mode, with depth and gain parameters optimized according to the specific characteristics of breast tissue, focusing on the region of interest (ROI). Grayscale ultrasound was performed first to identify the cross-section displaying the maximum lesion diameter. Lesion dimensions were accurately measured in two mutually perpendicular planes, and corresponding images were archived. Subsequently, CDFI and MV-Flow modes were sequentially activated to evaluate blood flow signals within the target lesion. Throughout the examination, excessive compression of the breast mass was strictly avoided to prevent artifact-induced distortion of hemodynamic signals. For CDFI, the flow velocity scale was set at 3.0–4.0 cm/s, whereas for MV-Flow, the scale was adjusted to 1.0–1.5 cm/s to optimize the visualization of microvascular perfusion. For MV-Flow, the transmission center frequency was maintained at approximately 9–10 MHz to ensure sufficient sensitivity to microvascular flow while preserving spatial resolution. In addition, velocity scales were separately optimized according to each modality’s technical specifications and clinical recommendations to ensure optimal visualization of detectable flow signals while minimizing artifacts. The cross-section exhibiting the most abundant blood flow was selected for dynamic observation and spectral validation to confirm the authenticity of detected flow signals, followed by image storage. In MV-Flow mode, the image was frozen at the cross-section with maximal vascularity, and the lesion boundary was manually delineated to calculate the quantitative Vascular Index (VI). Three repeated measurements were obtained, and the mean value was adopted for subsequent analysis.

#### 2.2.3 Ultrasound image interpretation

The examining radiologist performed a comprehensive assessment of grayscale morphological features and hemodynamic patterns for each lesion, assigning a BI-RADS classification in accordance with the criteria established by the American College of Radiology (ACR).

Additionally, two independent radiologists with equivalent professional qualifications, who were blinded to the clinical and pathological outcomes, retrospectively interpreted the stored CDFI and MV-Flow images using the Adler semi-quantitative grading system (18). The grading criteria were defined as follows: Grade 0: Absence of detectable blood flow signals within the lesion; Grade 1: Minimal vascularity, characterized by 1–2 punctate or slender rod-like flow signals, with the length of rod-like signals not exceeding half of the lesion diameter; Grade 2: Moderate vascularity, defined by 3–4 punctate flow signals or a single elongated flow signal penetrating the lesion, with the length reaching or exceeding half of the lesion diameter; Grade 3: Marked vascularity, identified by ≥ 5 punctate flow signals or two elongated flow signals penetrating the lesion, with the length reaching or exceeding half of the lesion diameter. In cases of inter-observer discrepancy in grading, a final consensus was reached through adjudication by the radiologist who performed the original ultrasound examinations.

### 2.3 Machine learning model construction and validation

#### 2.3.1 Feature selection

Six key variables were extracted from clinical and ultrasound datasets for model development, including: patient age (Age), maximum lesion diameter (Size), CDFI-derived Adler grade (Adler-CDFI), MV-Flow-derived Adler grade (Adler-MV-Flow), Vascular Index (VI), and BI-RADS classification.

#### 2.3.2 Data preprocessing and partitioning

The dataset comprising 101 breast lesions was randomly split into a training set and an independent test set at an 8:2 ratio to ensure an unbiased evaluation of model performance. Splitting was done at the lesion level; for patients with multiple lesions, lesions were assigned independently. All continuous variables were normalized using the min-max scaling method to transform data values into the range of (0, 1).

#### 2.3.3 Model training and comparison

On the training set, eight distinct binary classification models were constructed and trained using the AT_model_binary machine learning package. The models included Logistic Regression, Support Vector Machine (SVM), K-Nearest Neighbors (KNN), Random Forest, XGBoost, Naive Bayes, Decision Tree, and Neural Network. Hyperparameter optimization for each model was implemented via cross-validation techniques to enhance model generalization capability.

#### 2.3.4 Model performance evaluation

The predictive performance of each trained model was evaluated on the independent test set using the following metrics: Accuracy, Precision, Recall (i.e., Sensitivity), F1-Score, and the Area Under the Receiver Operating Characteristic Curve (ROC-AUC).

#### 2.3.5 Model interpretability analysis

For the optimal-performing model (K-Nearest Neighbors), SHapley Additive exPlanations (SHAP) was employed to conduct interpretability analysis. The mean SHAP value for each feature was calculated to quantify its global importance in predictive decision-making. Furthermore, SHAP summary plots were generated to visualize the direction and magnitude of each feature’s impact on individual prediction outcomes.

### 2.4 Statistical analysis

Statistical analyses were performed using SPSS version 25.0 and R version 4.5.1 software. Normality of continuous variables was assessed using appropriate tests. Normally distributed continuous data were expressed as mean ± standard deviation (Mean ± SD), and comparisons between two groups were performed using the independent samples t-test. Non-normally distributed continuous data were presented as median (interquartile range) [M (P25, P75)], and between-group comparisons were conducted using the Mann–Whitney U test.

Inter-observer agreement for Adler grading of CDFI and MV-Flow images was evaluated using the weighted Kappa coefficient. Receiver Operating Characteristic (ROC) curves were plotted to assess the diagnostic efficacy of individual and combined variables in differentiating benign from malignant breast lesions, with corresponding Sensitivity, Specificity, and AUC values calculated. Pearson’s correlation coefficient was used to analyze the linear correlation between continuous variables. All statistical tests were two-tailed, and a P-value < 0.05 was considered statistically significant.

## 3 Results

### 3.1 Demographic and clinical characteristics

**Figure 1:**
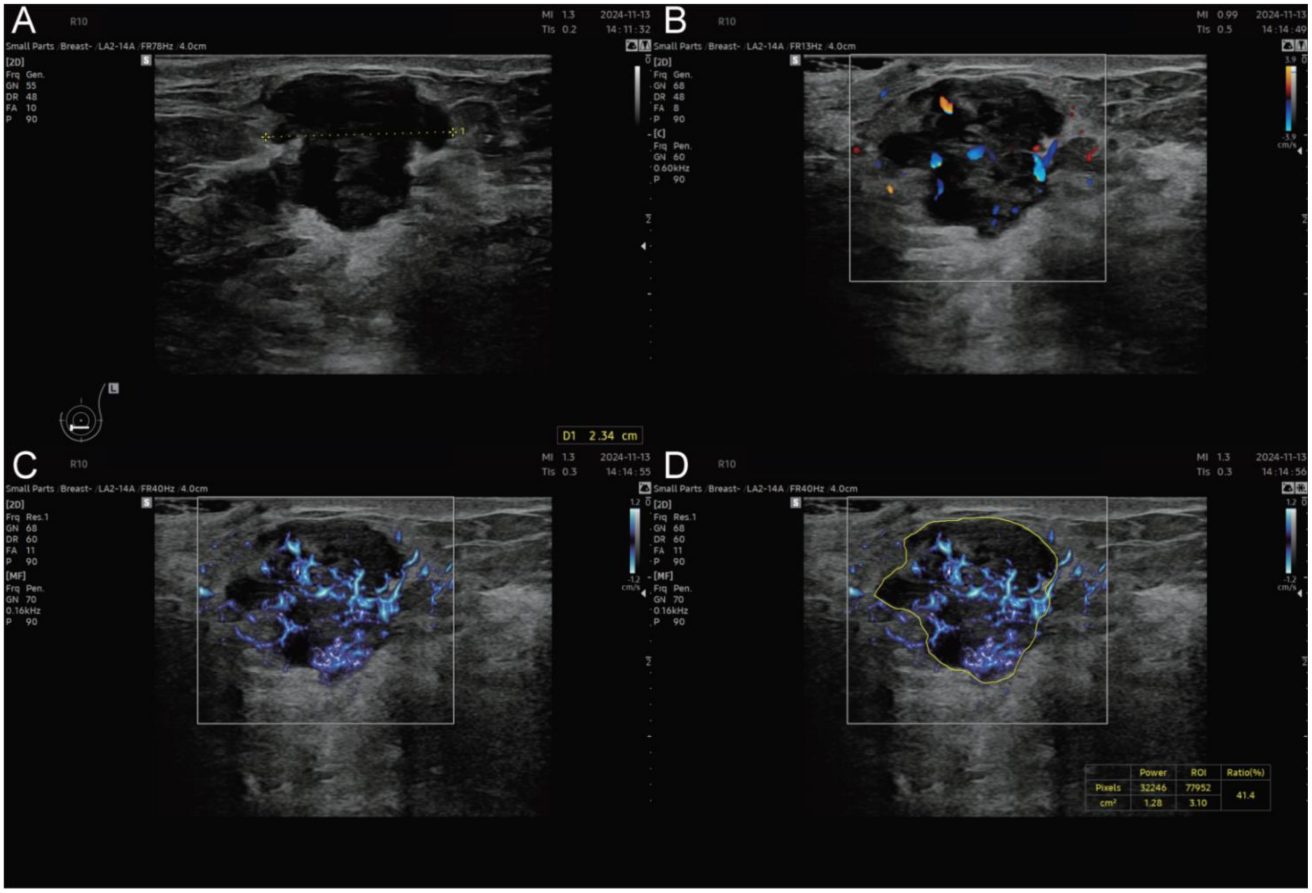
Grayscale ultrasound, CDFI, and MV-Flow images of invasive breast carcinoma in the left breast of a 56-year-old female. **(A)** Grayscale ultrasound features: irregular morphology, ill-defined margins, heterogeneous internal echogenicity, posterior acoustic enhancement, and anterior protrusion into the subcutaneous fat layer. **(B)** CDFI demonstrates Adler grade 3 vascularity. **(C)** MV-Flow reveals Adler grade 3 vascularity. **(D)** A quantitative Vascular Index **(VI)** of 41.4 is obtained in MV-flow mode. The final ultrasound diagnosis was categorized as BI-RADS 4C.

A total of 101 target lesions from 79 female patients were ultimately enrolled in this study, with the patients’ age ranging from 19 to 84 years and a mean age of 44.49 ± 14.29 years. Among these lesions, 44 were pathologically confirmed via percutaneous biopsy or surgical resection, including 20 malignant neoplasms and 24 benign lesions; detailed pathological diagnoses are summarized in **Table 1**. The remaining 57 lesions were classified as benign based on the absence of significant morphological alterations during a 12-month imaging follow-up. Notably, the mean age of patients with malignant lesions was significantly higher than that of those with benign lesions (57.15 ± 12.95 *vs.* 39.49 ± 11.78, t = 0.98, P < 0.001). Representative grayscale ultrasound, CDFI, and MV-Flow images of invasive breast carcinoma are presented in **Figure 1**.

**Table 1:** The final results of breast lesions confirmed by puncture or surgical pathology.

Malignant tumor	N	Benign tumor	N
Invasive carcinoma	14	Fibroadenoma	11
Invasive carcinoma with intermediate-grade ductal carcinoma in situ	1	Intraductal papilloma	3
Invasive solid papillary carcinoma	1	Tubular adenoma	1
High-grade ductal carcinoma in situ	3	Adenosis	6
Apocrine ductal carcinoma in situ	1	Sclerosing adenosis	2
		Benign phyllodes tumor	1
Total	20		24

### 3.2 Ultrasound features and outcomes of target lesions

Complete ultrasound datasets were available for all 101 breast lesions. The maximum diameter of the lesions, as measured by ultrasound, ranged from 0.5 to 4.8 cm, with a median value of 1.10 (0.80, 1.70) cm. The Adler grading results for CDFI vascularity assessed independently by two radiologists were as follows: Grade 0 (41 *vs.* 47 lesions), Grade 1 (33 *vs.* 21 lesions), Grade 2 (9 *vs.* 11 lesions), and Grade 3 (18 *vs.* 22 lesions). The inter-observer agreement for CDFI grading was relatively moderate, with a weighted kappa coefficient of 0.51 (95% confidence interval [CI]: 0.40–0.62). In contrast, the Adler grading results for MV-Flow vascularity were: Grade 0 (25 *vs.* 25 lesions), Grade 1 (29 *vs*. 21 lesions), Grade 2 (22 *vs.* 14 lesions), and Grade 3 (25 *vs.* 41 lesions), which yielded a significantly improved inter-observer agreement, with a weighted kappa coefficient of 0.68 (95% CI: 0.59–0.77). The Vascular Index (VI) measured in MV-Flow mode ranged from 0.0 to 60.6, with a median of 4.80 (0.40, 14.90). For malignant lesions, the VI ranged from 1.0 to 60.6, with a median of 20.25 (6.88, 37.80) and an overall rank mean of 77.18, which was significantly higher than those of benign lesions (VI range: 0.0–44.5; median: 3.10 [0.00, 9.80]; rank mean: 44.54) (U = 286.5, P < 0.001). Based on the comprehensive assessment of all ultrasound features, the final BI-RADS classifications of the lesions were determined as follows: 66 lesions categorized as BI-RADS 3, 13 as BI-RADS 4A, 12 as BI-RADS 4B, and 10 as BI-RADS 4C.

### 3.3 Diagnostic efficacy of MV-Flow-derived Adler grade and VI in differentiating benign and malignant breast tumors, alone and in combination

**Figure 2:**
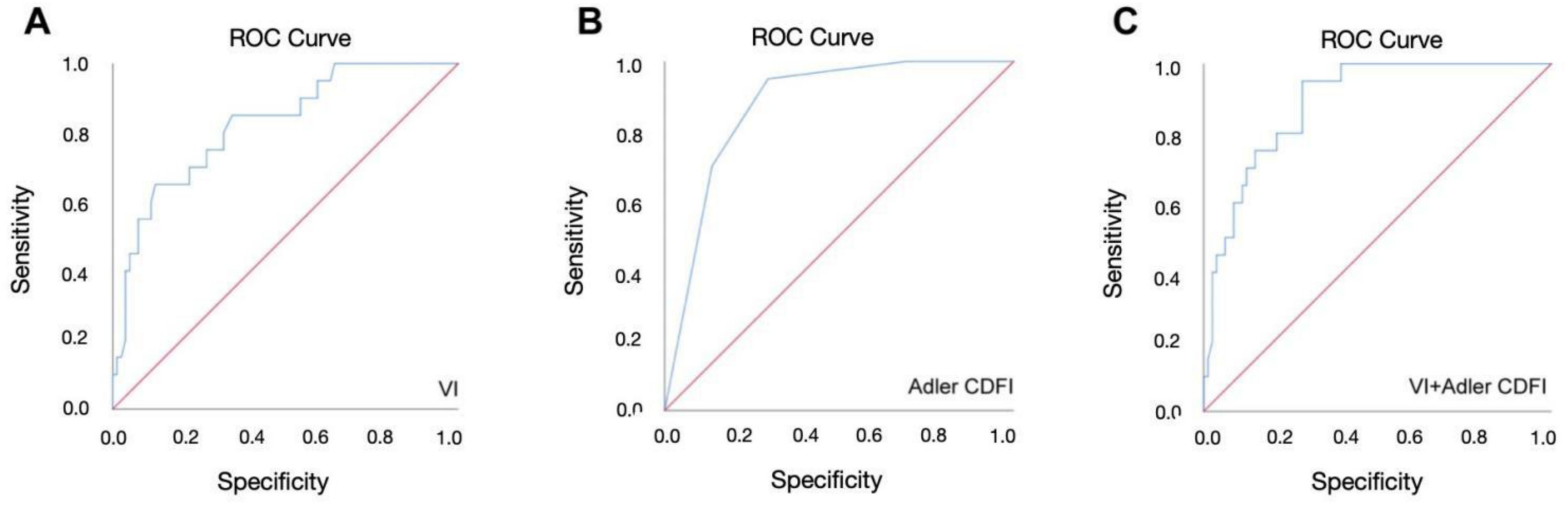
ROC curves for differentiating benign and malignant breast tumors using VI, Adler grade derived from MV-Flow, the combination of VI and Adler grade, and BI-RADS classification. **(A)** ROC curve of VI (AUC: 0.823; sensitivity: 65.0%; specificity: 87.7%); **(B)** ROC curve of MV-Flow-derived Adler grade (AUC: 0.874; sensitivity: 95.0%; specificity: 70.4%); **(C)** ROC curve of the combined VI and MV-Flow-derived Adler grade (AUC: 0.888; sensitivity: 95.0%; specificity: 71.6%).

Receiver Operating Characteristic (ROC) curves were plotted to evaluate the diagnostic efficacy of MV-Flow-derived Adler grade in identifying malignant breast tumors, as illustrated in **Figure 2**. When Adler Grade I was set as the cutoff value, the area under the ROC curve (AUC) was 0.874, with a sensitivity of 95.0% and a specificity of 70.4%. For VI as a standalone diagnostic indicator, the ROC curve analysis revealed that a cutoff value of 15.8 yielded an AUC of 0.823, with a sensitivity of 65.0% and a specificity of 87.7%. Notably, the combined application of MV-Flow-derived Adler grade and VI achieved a higher diagnostic performance, with an AUC of 0.888, a sensitivity of 95.0%, and a specificity of 71.6%.

### 3.4 Baseline patient characteristics and variable distribution

**Figure 3:**
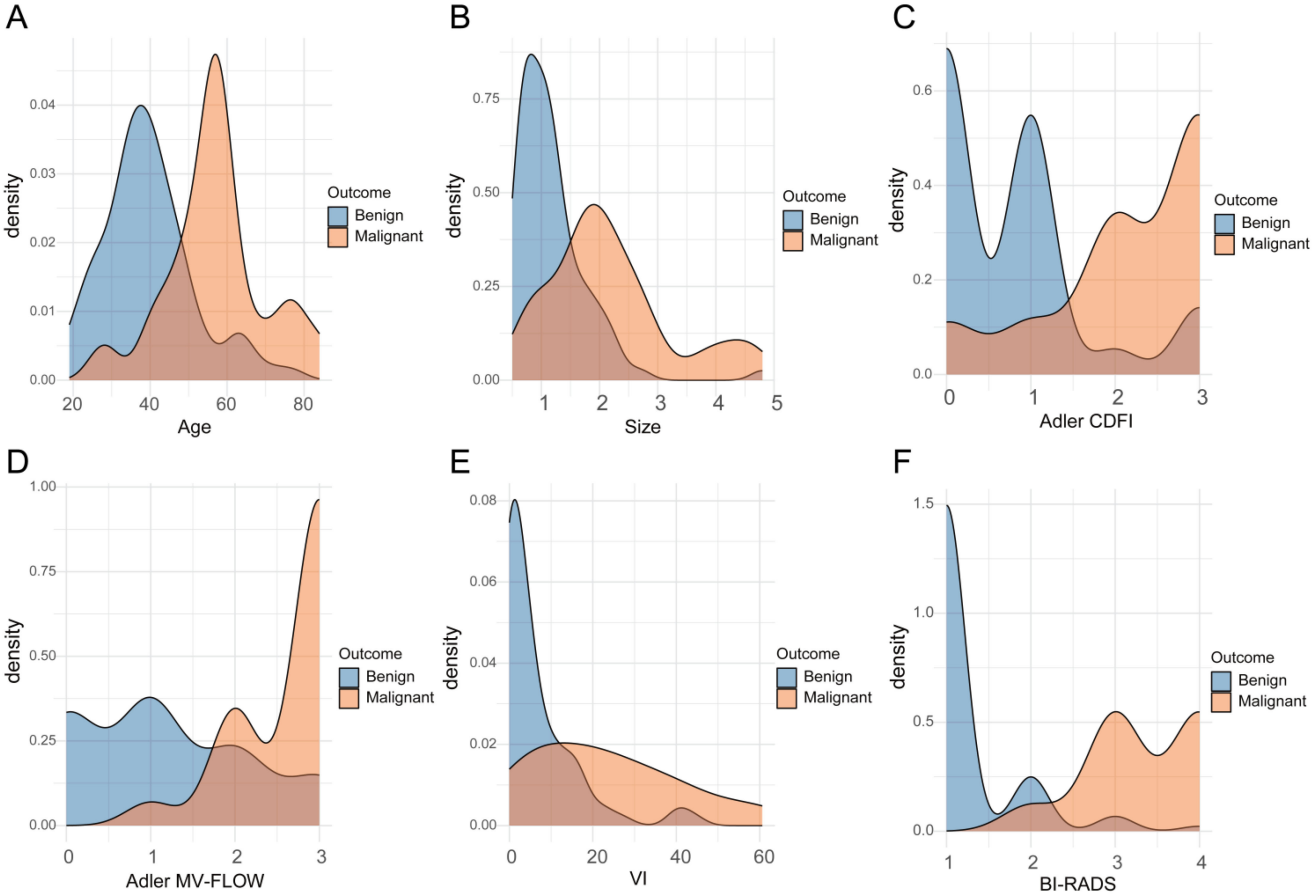
Distribution differences of the six key predictive variables incorporated into the machine learning models between benign and malignant breast tumor groups. Kernel density estimation curves of **(A)** age, **(B)** tumor size, **(C)** CDFI-derived Adler grade (Adler-CDFI), **(D)** MV-Flow-derived Adler grade (Adler-MV-Flow), **(E)** Vascular Index (VI), and **(F)** BI-RADS classification. The blue curve represents pathologically confirmed benign tumors, and the orange curve represents pathologically confirmed malignant tumors. The vertical axis denotes the kernel density estimate, reflecting the central tendency of data distribution.

The density curves depicting the distribution of key variables between benign and malignant subgroups are presented in **Figure 3**. The age distribution of the malignant group exhibited a slight rightward shift (**Figure 3A**). In addition, tumor size showed a broader distribution with higher density in the high-value range among malignant lesions (**Figure 3B**). Most strikingly, the density curve of VI—a hallmark parameter of microvascular imaging—displayed a prominent rightward shift with peak concentration in the higher value interval for malignant tumors. This finding clearly indicates that malignant neoplasms are characterized by more abundant and disorganized angiogenesis, directly validating the differential diagnostic value of MV-Flow technology (**Figures 3C–E**). Furthermore, the distribution of benign and malignant tumors was consistent with BI-RADS classification stratification (**Figure 3F**).

### 3.5 Correlation analysis between ultrasound features and breast tumor malignancy

**Figure 4:**
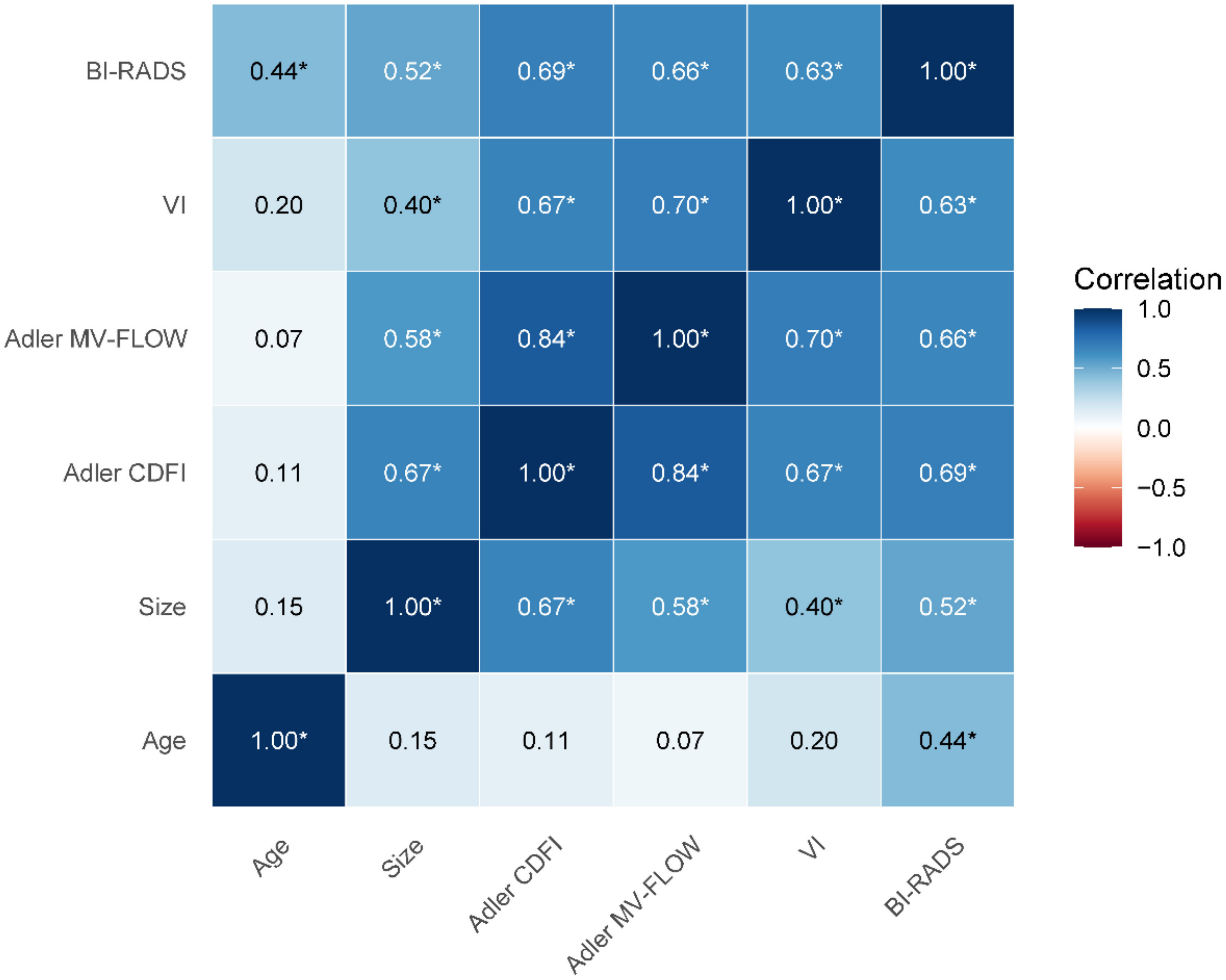
Pearson correlation matrix heatmap of the predictive variables in the machine learning models. Each cell in the matrix represents the correlation strength between the row variable and the column variable, with the corresponding value labeled. The correlation magnitude is visualized using a color gradient ranging from dark blue (strong positive correlation, r = +1.0) to white (no correlation, r = 0.0) and further to dark red (strong negative correlation, r = −1.0). Asterisks (*) in the upper right corner of the values indicate statistically significant correlations (P < 0.05).

Correlation analysis was performed on the predictive variables included in the machine learning models, with the results visualized in **Figure 4**. The variables exhibited distinct correlation patterns. Specifically, a strong positive correlation was observed between CDFI-derived Adler grade and MV-Flow-derived Adler grade (r = 0.84), both of which reflect tumor vascularity. BI-RADS classification showed a moderate positive correlation with MV-Flow-derived VI (r = 0.63), suggesting a meaningful association between radiologically assessed malignant risk and objectively measured microvascular density. Tumor size was also positively correlated with CDFI-derived Adler grade (r = 0.67), MV-Flow-derived Adler grade (r = 0.58), VI (r = 0.40), and BI-RADS classification (r = 0.52). In contrast, patient age showed weak correlations (r < 0.20) with all variables except BI-RADS classification, indicating that age may serve as a relatively independent predictive factor.

### 3.6 Evaluation and comparison of the diagnostic performance of ultrasound feature-based models

**Figure 5:**
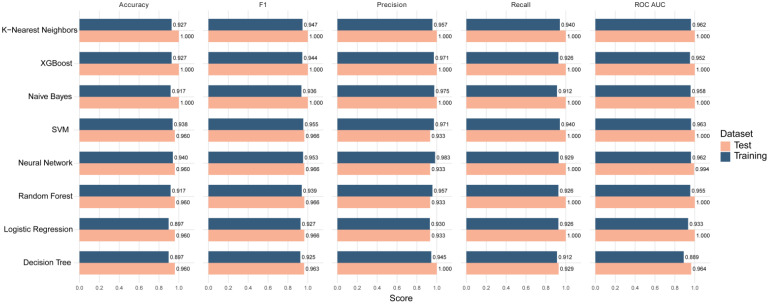
Performance comparison of different machine learning models on the training set and test set. The figure displays five evaluation metrics for each model on the training set (dark blue bars) and independent test set (light orange bars): Accuracy, F1-Score, Precision, Recall, and ROC-AUC.

Based on six variables—age, tumor size, CDFI-derived Adler grade, MV-Flow-derived Adler grade, VI, and BI-RADS classification—this study systematically evaluated the performance of eight machine learning models in differentiating benign and malignant breast tumors. As shown in **Figure 5**, all models exhibited excellent performance on the training set (with most metrics approaching 1.0), whereas their performance diverged significantly on the independent test set, indicating a certain degree of overfitting in some models. On the training set, the XGBoost and K-Nearest Neighbors (KNN) models demonstrated the most robust comprehensive performance, with an accuracy of 0.927 each and F1-scores of 0.944 and 0.947, respectively. The Support Vector Machine (SVM) and Neural Network models also yielded favorable results, with accuracies of 0.938 and 0.940, respectively. Notably, the Random Forest and Logistic Regression models achieved relatively lower performance on the training set. Given the divergence in generalization across models, the K-Nearest Neighbors model was selected as optimal based on its balanced test-set performance. A comprehensive analysis of all metrics revealed that the tree-based ensemble algorithm (K-Nearest Neighbors) achieved the optimal balance between discriminative power and generalization ability, demonstrating its efficacy in integrating clinical and microvascular imaging features to construct a high-performance diagnostic prediction model.

### 3.7 Model interpretability analysis

**Figure 6:**
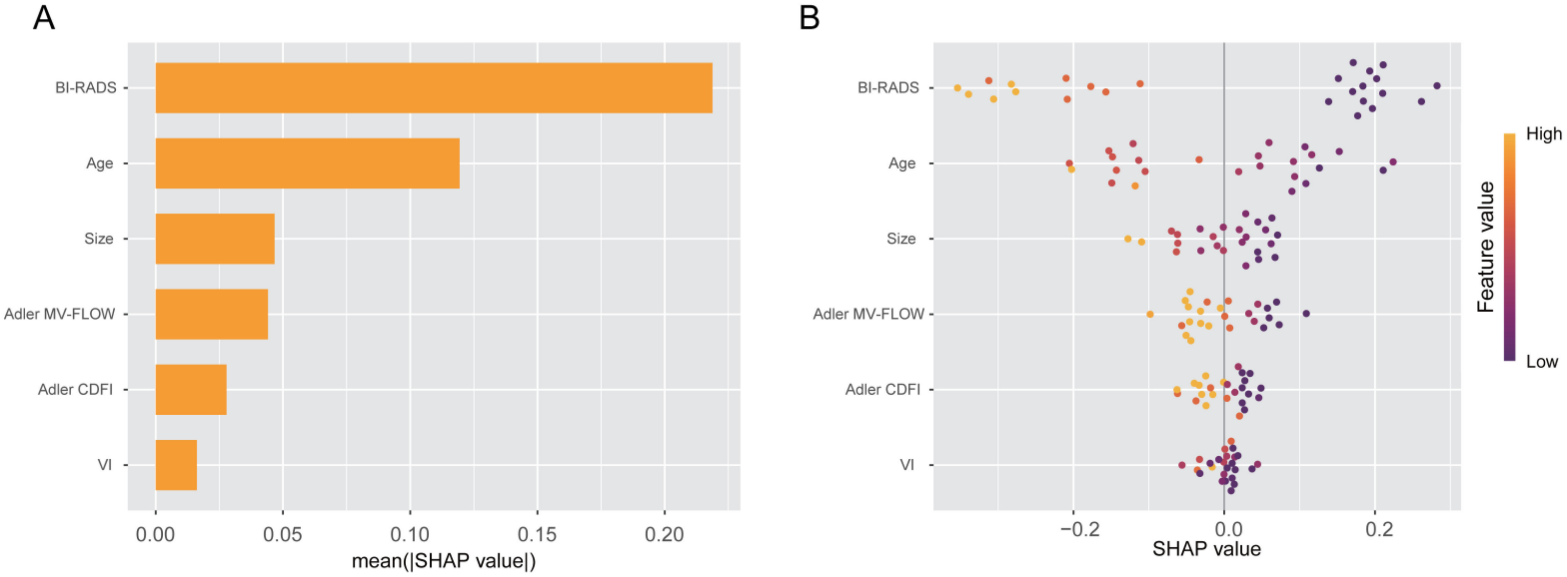
Interpretability analysis of the optimal model (K-nearest neighbors). **(A)** Feature importance ranking plot: the values represent the average magnitude of each feature’s impact on the model’s predictive output; **(B)** SHAP summary plot: the effect of feature values (color gradient from purple/low to yellow/high) on the direction of model prediction (SHAP value on the horizontal axis), where positive SHAP values shift predictions toward benignancy and negative values shift predictions toward malignancy.

On the basis of model performance evaluation, SHapley Additive exPlanations (SHAP) was employed to elucidate the decision-making rationale of the optimal model (K-Nearest Neighbors), with the results presented in **Figure 6**. Feature importance analysis indicated that among the six incorporated variables, BI-RADS classification had the highest global importance, followed by patient age, while VI had the lowest global importance. The SHAP summary plot further delineated the specific direction and magnitude of each variable’s impact on model output. A positive SHAP value indicates a predictive tendency toward benignancy, whereas a negative SHAP value indicates a tendency toward malignancy. Specifically, BI-RADS classification emerged as the most impactful predictor: higher BI-RADS categories (represented by red dots) were associated with larger SHAP values, strongly driving the model to predict malignancy, which is highly consistent with clinical guidelines. Age was the second most contributory feature, with advanced age (red dots) also positively correlated with an elevated risk of malignant prediction. Tumor size exerted a similar effect, with larger tumor dimensions tending to increase the probability of malignant classification. It is noteworthy that despite its low global importance, the SHAP value distribution of VI revealed that lower VI values (blue dots) were primarily associated with negative SHAP values, suggesting that reduced microvascular density is linked to benign prediction in the constructed model. In contrast, the impact direction of higher VI values (red dots) was relatively dispersed, which may be attributed to the overlap or interaction of its information with that of strong predictive features such as BI-RADS classification, thereby partially masking its independent contribution. In summary, the interpretability analysis confirmed that the model’s decisions are primarily dependent on key clinical and imaging features including BI-RADS classification and age, with the impact direction aligning with medical logic, which enhances the reliability and clinical acceptability of the model.

## 4 Discussion

In recent years, remarkable advances in ultrasonic Doppler technology have enabled the refined assessment of vascularity in breast tumors. Emerging evidence indicates that Doppler manifestations indicative of malignancy, such as abundant blood supply, central vascular distribution, and penetrating or branching vessels facilitate the differential diagnosis of benign and malignant breast lesions (13, 19). Color Doppler Flow Imaging (CDFI) is currently the most widely adopted vascular imaging modality in clinical practice; however, its inherent limitations in detecting low-volume and slow-flow blood signals result in substantial overlap of hemodynamic features between benign and malignant tumors (20). In contrast, Microvascular Flow Imaging (MV-Flow) represents a representative cutting-edge technique, exhibiting superior sensitivity in microvascular detection and holding great potential for enhancing the diagnostic accuracy of breast malignancy (21). In the present study, 41 breast lesions showed no detectable blood flow on CDFI, whereas 15 of these lesions were upgraded to Adler Grade 1 and 1 lesion was classified as Adler Grade 3 (marked vascularity) on MV-Flow. These findings further corroborate the high sensitivity of MV-Flow for microvascular perfusion, enabling a more faithful reflection of intralesional hemodynamics. Moreover, consistent with previous research, MV-Flow demonstrated greater efficacy than CDFI in identifying high-grade vascularity in malignant tumors. Among the 20 malignant breast lesions, CDFI yielded Adler Grade 0 in 2 lesions, Grade 1 in 2 lesions, Grade 2 in 6 lesions, and Grade 3 in 10 lesions, while MV-Flow predominantly detected high-grade vascularity in these malignant neoplasms.

The Adler semi-quantitative grading system provides a semi-quantitative assessment of tumor vascularity, compensating for the limitation of grayscale ultrasound, which only yields morphological information. This grading system plays a pivotal role in differentiating benign from malignant breast lesions, evaluating tumor biological behavior, and monitoring the efficacy of chemotherapy (22–24). Nevertheless, its heavy reliance on operator expertise and the subjective nature of vascularity assessment have to some extent compromised the consistency of diagnosing breast lesions. In this study, two independent radiologists performed Adler grading of breast lesion vascularity, and the results indicated significantly improved inter-observer agreement for MV-Flow compared with CDFI. This finding suggests that MV-Flow is a more robust technique suitable for routine clinical application. Furthermore, the enhanced sensitivity of MV-Flow allows for the visualization of more microvascular signals, which substantially elevates inter-observer consistency (with the weighted kappa coefficient increasing from 0.51 to 0.68). This observation is highly consistent with the review by Park and Seo, which emphasized that emerging Doppler technologies such as MV-Flow possess inherent advantages in detecting slow blood flow (21). It is noteworthy, however, that breast tumors constitute a highly heterogeneous group of diseases, with overlapping hemodynamic features across benign and malignant subtypes. In the current study, MV-Flow-derived Adler grading exhibited relatively low specificity (70.4%) in diagnosing breast malignancy, indicating that it cannot be used as a standalone diagnostic criterion. For instance, one case of invasive carcinoma in our cohort only presented with Adler Grade 1 vascularity, with a maximum diameter of 0.7 cm; this finding may be attributed to the small tumor size and the absence of a well-established abundant vascular network. Conversely, two cases of intraductal papilloma, one case of sclerosing adenosis, and one case of benign phyllodes tumor were classified as Adler Grade 3. This phenomenon may be explained by the presence of fibrovascular cores and stromal cell proliferation, which stimulate angiogenesis and thus lead to abundant intralesional blood flow.

The Vascular Index (VI) is a quantitative analytical parameter derived from MV-Flow technology, which quantifies the degree of vascularity by calculating the ratio of blood flow pixels to the total pixels within the region of interest (ROI), or the ratio of blood flow area to the total ROI area. VI holds promise for providing a more objective assessment of tumor vascularization. In this study, the median VI value of malignant breast tumors was significantly higher than that of benign lesions. Density distribution plots revealed a prominent rightward shift of the VI curve peak in the malignant group, with values concentrated in the higher interval, clearly indicating that malignant tumors are characterized by more abundant and disorganized angiogenesis. This finding is consistent with previous research, although substantial variations in VI cutoff values have been reported across different studies, which may be attributed to differences in study populations and disease spectra (25). In the current cohort, a VI cutoff value of 15.8 achieved favorable discriminative efficacy (AUC = 0.823) and high specificity (87.7%) in differentiating benign and malignant breast lesions, which could effectively reduce the number of unnecessary percutaneous biopsies in clinical practice. However, the relatively low sensitivity (65.0%) of VI may pose a risk of missed diagnoses. Therefore, in this study, we combined the semi-quantitative Adler grading with the quantitative VI parameter. The integrated application of these two indices significantly enhanced the overall diagnostic efficacy for breast malignancy (AUC = 0.888), achieving a marked improvement in sensitivity (95.0%) without excessive sacrifice of specificity (71.6%). The implementation of this comprehensive diagnostic model will substantially improve the differential diagnosis of benign and malignant breast tumors, providing robust evidence to support the clinical application of MV-Flow technology.

Building on the advantages of MV-Flow in visualizing the microvasculature of breast tumors, this study innovatively integrated machine learning models into the comprehensive analysis of multi-parametric ultrasound features. Variable distribution and correlation analyses demonstrated that the malignant group had significantly higher VI values than the benign group, which is consistent with the findings of Zhang et al., who reported that VI measured by three-dimensional microvascular imaging facilitates the differentiation of benign and malignant breast lesions (25). Furthermore, a strong positive correlation was observed between CDFI-derived Adler grade and MV-Flow-derived Adler grade (r = 0.84), while a moderate positive correlation was noted between BI-RADS classification and VI (r = 0.63). This correlation pattern suggests that although there is an overlap of information captured by different vascular assessment methods, the correlation between BI-RADS classification—a composite index integrating morphological and hemodynamic features and quantitative VI confirms the biological association between morphological abnormalities and angiogenesis. This finding aligns with the classic paradigm that breast cancer is a vascular-dependent neoplasm (26).

The superior performance of machine learning models constitutes a core finding of this study. On the independent test set, the K-Nearest Neighbors (KNN) model exhibited the most optimal and balanced performance, with its advantages lying in its ability to effectively handle nonlinear relationships between features and maintain sensitivity to local data patterns. This stands in contrast to traditional statistical methods (e.g., Logistic Regression), which often assume linear relationships between variables. The results of this study are consistent with the growing trend of applying machine learning to improve diagnostic accuracy in medical imaging; for example, similar algorithms have demonstrated immense potential in CT screening of lung nodules and dermoscopic image analysis (16, 17). The performance discrepancy between the training set and the test set observed in some models (e.g., Random Forest) directly reveals the issue of overfitting, underscoring the importance of selecting algorithms with strong generalization ability (e.g., KNN) and validating model performance using an independent test set, especially when working with limited sample sizes. The results showed that the KNN model constructed based on six features achieved an accuracy of 0.927 and an F1-score of 0.947 on the independent test set, with its diagnostic efficacy surpassing that of single hemodynamic parameters and even their simple combinations. This implies that the paradigm of ultrasound-based breast tumor diagnosis may evolve from subjective interpretation of limited features by clinicians to in-depth mining of multi-dimensional information and intelligent decision-making with the aid of artificial intelligence algorithms.

Model interpretability analysis serves as a critical bridge connecting the “black box” of artificial intelligence with clinical cognition. SHAP-based analysis revealed that BI-RADS classification was the most important feature in the model’s decision-making process, which is closely related to the high generality of this classification system, as it integrates multiple key diagnostic indicators. This finding not only verifies the high consistency between the model’s decision logic and current clinical guidelines but also greatly enhances clinicians’ trust in the model’s outputs (27). Patient age emerged as the second most important feature, and its contribution is fully consistent with the epidemiological evidence from global cancer statistics, which indicates that the incidence of breast cancer increases significantly with age (1). Notably, despite its relatively low global importance, the SHAP value distribution of VI still revealed its potential association with benign prediction, suggesting the possibility that VI may provide incremental diagnostic information in specific scenarios. This interpretability analysis provides a transparent window for understanding the model’s decision-making mechanism, which is an indispensable step for artificial intelligence-assisted diagnostic tools to translate into clinical practice.

The present study has several limitations. First, as a single-center study, there may be selection bias in patient enrollment. Future research should conduct multicenter, large-sample external validation to rigorously evaluate the generalizability and robustness of the model. Although rigorous splitting and regularization were used to reduce overfitting, the limited malignant cases (n=20) warrant caution. Future multicenter expansion is needed to confirm generalizability. Furthermore, a portion of benign lesions were defined by 12-month imaging stability rather than pathology, potentially misclassifying indolent malignancies; this reflects real-world practice but warrants caution in interpreting benign-class metrics. Second, the extraction of ultrasound features still involves a certain degree of subjectivity. Although MV-Flow has improved the consistency of vascularity assessment, operator dependence remains a challenge for its widespread clinical implementation. Future studies could explore the use of deep learning techniques for end-to-end automated feature extraction and classification, with the aim of further reducing human-induced variability and improving standardization. Third, the model constructed in this study is based on conventional ultrasound and MV-Flow parameters. Future research may consider incorporating more radiomic features, molecular pathological markers, and even multi-modal imaging data (e.g., mammography, magnetic resonance imaging) to build a more comprehensive and powerful prediction system, thereby achieving truly precise diagnosis and individualized risk assessment. Fourth, our flow characterization relied on VI and Adler grades as representative quantitative and semi-quantitative descriptors; future models could include additional microvascular parameters (e.g., vessel tortuosity, branching patterns) for deeper vascular phenotyping. Fifth, train-test partitioning was performed at the lesion level; future studies with larger cohorts should adopt patient-level splitting to fully eliminate within-patient dependencies.

In conclusion, this study not only further confirms the significant advantages of MV-Flow technology in the assessment of breast tumor vascularity but also innovatively demonstrates the enormous potential and clinical translational value of machine learning models in integrating multi-parametric ultrasound data to achieve accurate differential diagnosis. The combination of advanced vascular imaging technology with interpretable artificial intelligence algorithms is expected to evolve into a reliable intelligent tool to assist ultrasound clinicians in clinical decision-making in the future. This innovative “imaging technology + artificial intelligence” model holds promise for significantly improving the objectivity, efficiency, and accuracy of diagnosis, optimizing clinical diagnosis and treatment pathways, and ultimately benefiting a broad population of patients. Future research directions should focus on large-scale clinical validation, technological automation and standardization, and multi-dimensional information fusion to advance this field to a higher level.

## Data Availability

The original contributions presented in the study are included in the article/Supplementary Material. Further inquiries can be directed to the corresponding author.
